# Substance Abuse Amongst Adolescents: An Issue of Public Health Significance

**DOI:** 10.7759/cureus.31193

**Published:** 2022-11-07

**Authors:** Aditi Nath, Sonali G Choudhari, Sarika U Dakhode, Asmita Rannaware, Abhay M Gaidhane

**Affiliations:** 1 School of Epidemiology and Public Health, Jawaharlal Nehru Medical College, Datta Meghe Institute of Medical Sciences, Wardha, IND; 2 School of Epidemiology and Public Health; Community Medicine, Jawaharlal Nehru Medical College, Datta Meghe Institute of Medical Sciences, Wardha, IND; 3 Department of Community Medicine, Dr. Panjabrao Deshmukh Memorial Medical College, Amravati, IND

**Keywords:** narcotic drugs and psychotropic substances act, drug and substance abuse, stimulant abuse, adolescent addiction, adolescents' health

## Abstract

Adolescence is a crucial time for biological, psychological, and social development. It is also a time when substance addiction and its adverse effects are more likely to occur. Adolescents are particularly susceptible to the negative long-term effects of substance use, including mental health illnesses, sub-par academic performance, substance use disorders, and higher chances of getting addicted to alcohol and marijuana. Over the past few decades, there have been substantial changes in the types of illegal narcotics people consume. The present article deals with the review of substance abuse as a public health problem, its determinants, and implications seen among adolescents. A systematic literature search using databases such as PubMed and Google Scholar was undertaken to search all relevant literature on teenage stimulant use. The findings have been organized into categories to cover essential aspects like epidemiology, neurobiology, prevention, and treatment. The review showed that substance addiction among adolescents between 12 to 19 years is widespread, though national initiatives exist to support young employment and their development. Research on psychological risk factors for teenage substance abuse is vast, wherein conduct disorders, including aggression, impulsivity, and attention deficit hyperactivity disorder, have been mentioned as risk factors for substance use. Parents' attitudes toward drugs, alcohol, academic and peer pressure, stress, and physical outlook are key determinants. Teenage drug usage has a significant negative impact on users, families, and society as a whole. It was found that a lot has been done to provide correct intervention to those in need with the constant development of programs and rehabilitative centers to safeguard the delicate minds of youths and prevent them from using intoxicants. Still, there is much need for stringent policy and program guidelines to curb this societal menace.

## Introduction and background

Drug misuse is a widespread issue; in 2016, 5.6% of people aged 15 to 26 reported using drugs at least once [1]. Because alcohol and illegal drugs represent significant issues for public health and urgent care, children and adolescents frequently visit emergency rooms [2]. It is well known that younger people take drugs more often than older adults for most drugs. Drug usage is on the rise in many Association of Southeast Asian Nations, particularly among young males between the ages of 15 and 30 years [3]. According to the 2013 Global Burden of Disease report, drug addiction is a growing problem among teenagers and young people. Early substance use increases the likelihood of future physical, behavioral, social, and health issues [4]. Furthermore, recreational drug use is a neglected contributor to childhood morbidity and mortality [5]. One of the adverse outcomes of adolescent substance use is the increased risk of addiction in those who start smoking, drinking, and taking drugs before they are of 18 years. Moreover, most individuals with Substance Use Disorders begin using substances when they are young [6]. Substance use disorders amongst adolescents have long-term adverse health effects but can be mitigated with efficient treatment [7].

Childhood abuse is linked to suicidal thoughts and attempts. The particular mental behavior that mediates the link between childhood trauma and adult suicidal ideation and attempts is yet unknown. Recent studies show teens experiencing suicidal thoughts, psychiatric illness symptoms like anxiety, mood, and conduct disorders, and various types of child maltreatment like sexual abuse, corporal punishment, and emotional neglect that further leads to children inclining toward intoxicants [8]. Although teen substance use has generally decreased over the past five years, prolonged opioid, marijuana, and binge drinking use are still common among adolescents and young adults [9]. Drug-using students are more prone to commit crimes, including bullying and violent behavior. It has also been connected to various mental conditions, depending on the substance used. On the other hand, it has been linked to social disorder, abnormal behavior, and association with hostile groups [10]. Adolescent substance users suffer risks and consequences on the psychological, sociocultural, or behavioral levels that may manifest physiologically [11]. About 3 million deaths worldwide were caused by alcohol consumption alone. The majority of the 273,000 preventable fatalities linked to alcohol consumption are in India [12], which is the leading contributor. The United Nations Office on Drug and Crime conducted a national survey on the extent, patterns, and trends of drug abuse in India in 2003, which found that there were 2 million opiate users, 8.7 million cannabis users, and 62.5 million alcohol users in India, of whom 17% to 20% are dependent [13]. According to prevalence studies, 13.1% of drug users in India are under the age of 20 [14].

In India, alcohol and tobacco are legal drugs frequently abused and pose significant health risks, mainly when the general populace consumes them. States like Punjab and Uttar Pradesh have the highest rates of drug abuse, and the Indian government works hard to provide them with helpful services that educate and mentor them. This increases the burden of non-communicable illnesses too [15]. In addition, several substances/drugs are Narcotic and Psychotropic and used despite the act named ‘Narcotic Drugs and Psychotropic Substances Act, 1985. 

This review article sheds light on ‘substance abuse’ amongst adolescents as an issue of public health significance, its determinants, and its implications on the health and well-being of adolescents.

## Review

Methodology

**Figure 1:**
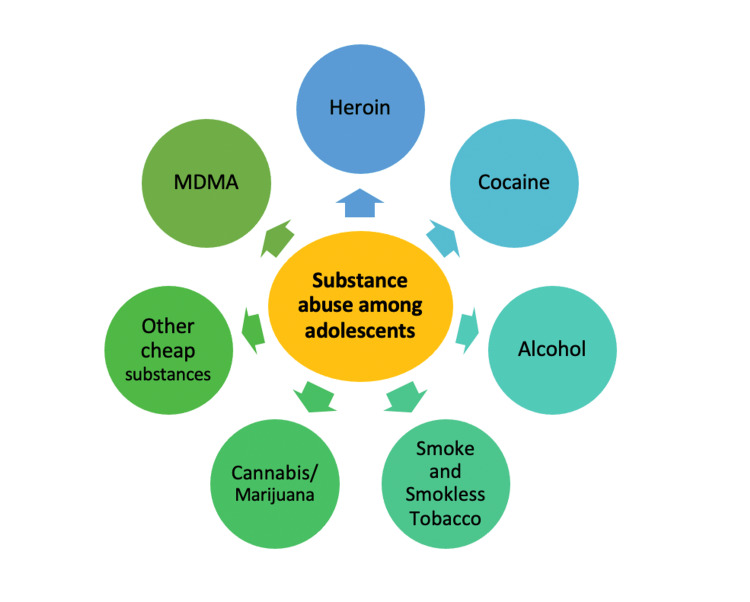
Key substances/stimulants abused by youth

The present article deals with the narrative review of substance abuse as a public health problem, its determinants, and implications seen among adolescents. A systematic literature search using databases such as PubMed and Google Scholar was undertaken to search all relevant literature on teenage stimulant use. The findings have been organized into categories to cover essential aspects like epidemiology, neurobiology, prevention, and treatment. Various keywords used under TiAb of PubMed advanced search were Stimulants, "Drug abuse", "Psychotropic substance", "Substance abuse", addiction, and Adolescents, teenage, children, students, youth, etc., including MeSH terms. Figure 1 shows the key substances used by youth.

Reasons for abuse

People may initially choose to take drugs for psychological and physical reasons. Psychological issues, including mental illness, traumatic experiences, or even general attitudes and ideas, might contribute to drug usage. Several factors can contribute to emotional and psychosocial stress, compelling one to practice drug abuse. It can be brought on by a loss of a job because of certain reasons, the death of a loved one, a parent's divorce, or financial problems. Even medical diseases and health problems can have a devastating emotional impact. Many take medicines to increase their physical stamina, sharpen their focus, or improve their looks.

Students are particularly prone to get indulged in substance abuse due to various reasons, like academic and peer pressure, the appeal of popularity and identification, readily available pocket money, and relatively easy accessibility of several substances, especially in industrial, urban elite areas, including nicotine (cigarettes) [16,17]. In addition, a relationship breakup, mental illness, environmental factors, self-medication, financial concerns, downtime, constraints of work and school, family obligations, societal pressure, abuse, trauma, boredom, curiosity, experimentation, rebellion, to be in control, enhanced performance, isolation, misinformation, ignorance, instant gratification, wide availability can be one of the reasons why one chooses this path [18].

Alcohol

The brain grows rapidly during adolescence and continues to do so until early adulthood, as is well documented. According to studies using structural magnetic resonance imaging, changes in cortical grey matter volume and thickness during development include linear and nonlinear transformations and increases in white matter volume and integrity. This delays the maturation of grey and white matter, resulting in poorer sustained attention [19]. Alcohol drinking excessively increases the likelihood of accidents and other harmful effects by impairing cognitive functions like impulse control and decision-making and motor functions like balance and hand-eye coordination [20]. Lower-order sensory motor regions of the brain mature first, followed by limbic areas crucial for processing rewards. The development of different brain regions follows different time-varying trajectories. Alcohol exposure has adversely affected various emotional, mental, and social functions in the frontal areas linked to higher-order cognitive functioning that emerge later in adolescence and young adulthood [21].

Smoking/e-cigarettes

The use of tobacco frequently begins before adulthood. A worryingly high percentage of schoolchildren between 13 and 15 have tried or are currently using tobacco, according to the global youth tobacco survey [22]. It is more likely that early adolescent cigarette usage will lead to nicotine dependence and adult cigarette use. Teenage smoking has been associated with traumatic stress, anxiety, and mood problems [23]. Nicotine usage has been associated with a variety of adolescent problems, including sexual risk behaviors, aggressiveness, and the use of alcohol and illegal drugs. High levels of impulsivity have been identified in adolescent smokers.

Additionally, compared to non-smokers, smoking is associated with a higher prevalence of anxiety and mood disorders in teenagers. Smoking is positively associated with suicidal thoughts and attempts [24]. Peer pressure, attempting something new, and stress management ranked top for current and former smokers [25]. Most teenagers say that when they start to feel down, they smoke to make themselves feel better and return to their usual, upbeat selves. Smoking may have varying effects on people's moods [26]. Teenagers who smoke seem more reckless, less able to control their impulses, and less attentive than non-smokers [27].

Cannabis/Marijuana

Marijuana is among the most often used illegal psychotropic substances in India and internationally. The prevalence of marijuana usage and hospitalizations related to marijuana are rising, especially among young people, according to current trends. Cannabis usage has been connected to learning, working memory, and attention problems. Cannabis has been shown to alleviate stress in small doses, but more significant amounts can cause anxiety, emotional symptoms, and dependence [28]. Myelination and synaptic pruning are two maturational brain processes that take place during adolescence and the early stages of adulthood. According to reports, these remodeling mechanisms are linked to efficient neural processing. They are assumed to provide the specialized cognitive processing needed for the highest neurocognitive performance. On a prolonged attentional processing test, marijuana usage before age 16 was linked to a shorter reaction time [29]. Cannabis use alters the endocannabinoid system, impacting executive function, reward function, and affective functions. It is believed that these disturbances are what lead to mental health problems [30].

MDMA (Ecstasy/Molly)

MDMA (3,4-methylenedioxy-methamphetamine) was a synthetic drug used legally in psychotherapy treatment throughout the 1970s, despite the lack of data demonstrating its efficacy. Molly, or the phrase "molecular," is typically utilized in powder form. Serotonin, dopamine, and norepinephrine are produced more significantly when MDMA is used. In the brain, these neurotransmitters affect mood, sleep, and appetite. Serotonin also causes the release of other hormones that may cause emotions of intimacy and attraction. Because of this, users might be more affectionate than usual and possibly develop ties with total strangers. The effects wear off three to six hours later, while a moderate dose may cause withdrawal symptoms to continue for a week. These symptoms include a decline in sex interest, a drop in appetite, problems sleeping, confusion, impatience, anxiety, sorrow, Impulsivity and violence, issues with memory and concentration, and insomnia are a few of them. Unsettlingly, it is rising in popularity in India, particularly among teenagers [31].

Opium 

In addition to being a top producer of illicit opium, India is a significant drug consumer. In India, opium has a long history. The most common behavioral changes are a lack of motivation, depression, hyperactivity, a lack of interest or concentration, mood swings or abrupt behavior changes, confusion or disorientation, depression, anxiety, distortion of reality perception, social isolation, slurred or slow-moving speech, reduced coordination, a loss of interest in once-enjoyed activities, taking from family members or engaging in other illegal activity [32]. Except for the chemical produced for medicinal purposes, it is imperative to prohibit both production and usage since if a relatively well-governed nation like India cannot stop the drug from leaking, the problem must be huge in scope [33].

Cocaine

Cocaine is a highly addictive drug that causes various psychiatric syndromes, illnesses, and symptoms. Some symptoms include agitation, paranoia, hallucinations, delusions, violence, and thoughts of suicide and murder. They may be caused by the substance directly or indirectly through the aggravation of co-occurring psychiatric conditions. More frequent and severe symptoms are frequently linked to the usage of cocaine in "crack" form. Cocaine can potentially worsen numerous mental diseases and cause various psychiatric symptoms.

Table 1 discusses the short- and long-term effects of substance abuse.

**Table 1:** Short-term and long-term effects of substances

Substance	Mode	Behavioral changes	Short-term physical effects	Long-term physical effects
Alcohol	Oral/drinking	Growingly aggressive self-disclosure racy sexual behavior [34].	Unsteady speech, Drowsiness, Vomiting, Diarrhea, Uneasy stomach, Headache, Breathing problems, Vision and hearing impairment, Faulty judgment, Diminution of perception and coordination, Unconsciousness, Anemia (loss of red blood cells), Coma, and Blackouts [35].	Unintentional injuries such as car crashes, falls, burns, drowning; Intentional injuries such as firearm injuries, sexual assault, and domestic violence; Increased on-the-job injuries and loss of productivity; increased family problems and broken relationships. Alcohol poisoning, High blood pressure, Stroke, and other heart-related diseases; Liver disease, Nerve damage, Sexual problems, Permanent damage to the brain [32]. Vitamin B_1_ deficiency can lead to a disorder characterized by amnesia, apathy, and disorientation. Ulcers, Gastritis (inflammation of stomach walls), Malnutrition, Cancer of the mouth and throat [35].
Cannabis	Smoked, Vaped, Eaten (mixed in food or brewed as tea)	Hallucinations, emotional swings, forgetfulness, Depersonalization, Paranoia, Delusions Disorientation. Psychosis, Bipolar illness, Schizophrenia [36].	Enhanced sensory perception and euphoria followed by drowsiness/relaxation; Slowed reaction time; problems with balance and coordination; Increased heart rate and appetite; problems with learning and memory; anxiety.	Mental health problems, Chronic cough, Frequent respiratory infections.
Cocaine (coke/crack)	Snorted, smoked, injected	Violence and hostility, paranoia and hallucinations, and monotonous or stereotyped simple conduct [37]. Suspiciousness anger\giddiness Irritability, and Impatience [38].	Narrowed blood vessels; enlarged pupils; increased body temperature, heart rate, and blood pressure; headache; abdominal pain and nausea; euphoria; increased energy, alertness; insomnia, restlessness; anxiety; erratic and violent behavior, panic attacks, paranoia, psychosis; heart rhythm problems, heart attack; stroke, seizure, coma.	Loss of sense of smell, nosebleeds, nasal damage and trouble swallowing from snorting; Infection and death of bowel tissue from decreased blood flow; Poor nutrition and weight loss; Lung damage from smoking.
Heroin	Injected, smoked, snorted	Exaggerated efforts to keep family members out of his or her room or being secretive about where he or she goes with friends; drastic changes in behavior and relationships with family and friends; sudden requests for money without a good reason; sudden disinterest in school activities or work; a drop in grades or work performance; a lack of energy and motivation; and lack of interest in clothes are all examples of these behaviors [39].	Euphoria; dry mouth; itching; nausea; vomiting; analgesia; slowed breathing and heart rate.	Collapsed veins; abscesses (swollen tissue with pus); infection of the lining and valves in the heart; constipation and stomach cramps; Liver or kidney disease; pneumonia.
MDMA	Swallowed, snorted	A state of exhilarated tranquility or peace greater sensitivity -More vigor both physically and emotionally -Increased intimacy and sociability -Relaxation -Bruxism -Empathy [40].	Lowered inhibition; enhanced sensory perception; increased heart rate and blood pressure; muscle tension; nausea; faintness; chills or sweating; sharp rise in body temperature leading to kidney failure or death.	Long-lasting confusion, Depression, problems with attention, memory, and Sleep; Increased anxiety, impulsiveness; Less interest in sex.
Cigarettes, Vaping devices, e-cigarettes, Cigars, Bidis, Hookahs, Kreteks	Smoked, snorted, chewed, vaporized	Hyperactivity Inattention [41]. Anxiety, Tension, enhanced emotions, and focus lower rage and stress, relax muscles, and curbs appetite [42].	Increased blood pressure, breathing, and heart rate; Exposes lungs to a variety of chemicals; Vaping also exposes the lungs to metallic vapors created by heating the coils in the device.	Greatly increased risk of cancer, especially lung cancer when smoked and oral cancers when chewed; Chronic bronchitis; Emphysema; Heart disease; Leukemia; Cataracts; Pneumonia [43].

Other cheap substances (*sasta nasha*) used in India

India is notorious for phenomena that defy comprehension. People in need may turn to readily available items like Iodex sandwiches, fevibond, sanitizer, whitener, etc., for comfort due to poverty and other circumstances to stop additional behavioral and other changes in youth discouragement is necessary [42-44]. 

Curbing drug abuse amongst youth

Seventy-five percent of Indian households contain at least one addict. The majority of them are fathers who act in this way due to boredom, stress from their jobs, emotional discomfort, problems with their families, or problems with their spouses. Due to exposure to such risky behaviors, children may try such intoxicants [45]. These behaviors need to be discouraged because they may affect the child's academic performance, physical growth, etc. The youngster starts to feel depressed, lonely, agitated and disturbed. Because they primarily revolve around educating students about the dangers and long-term impacts of substance abuse, previous attempts at prevention have all been ineffective. To highlight the risks of drug use and scare viewers into abstaining, some programs stoked terror. The theoretical underpinning of these early attempts was lacking, and they failed to consider the understanding of the developmental, social, and other etiologic factors that affect teenage substance use. These tactics are based on a simple cognitive conceptual paradigm that says that people's decisions to use or abuse substances depend on how well they are aware of the risks involved. More effective contemporary techniques are used over time [46]. School-based substance abuse prevention is a recent innovation utilized to execute changes, including social resistance skills training, normative education, and competence enhancement skills training.

Peer pressure makes a teenager vulnerable to such intoxicants. Teenagers are often exposed to alcohol, drugs, and smoking either because of pressure from their friends or because of being lonely. Social resistance training skills are used to achieve this. The pupils are instructed in the best ways to steer clear of or manage these harmful situations. The best method to respond to direct pressure to take drugs or alcohol is to know what to say (i.e., the specific content of a refusal message) and how to say it. These skills must be taught as a separate curriculum in every school to lower risk. Standard instructional methods include lessons and exercises to dispel misconceptions regarding drug usage's widespread use. 

Teenagers typically exaggerate how common it is to smoke, drink, and use particular substances, which could give off the impression that substance usage is acceptable. We can lessen young people's perceptions of the social acceptability of drug use by educating them that actual rates of drug usage are almost always lower than perceived rates of use. Data from surveys that were conducted in the classroom, school, or local community that demonstrate the prevalence of substance use in the immediate social setting may be used to support this information. If not, this can be taught using statistics from national surveys, which usually show prevalence rates that are far lower than what kids describe.

The role social learning processes have in teen drug use is recognized by competency-improvement programs, and there is awareness about how adolescents who lack interpersonal and social skills are more likely to succumb to peer pressure to use drugs. These young people might also be more inclined to turn to drug usage instead of healthier coping mechanisms. Most competency enhancement strategies include instruction in many of the following life skills: general problem-solving and decision-making skills, general cognitive abilities for fending off peer or media pressure, skills for enhancing self-control, adaptive coping mechanisms for reducing stress and anxiety through the use of cognitive coping mechanisms or be behavioral relaxation techniques, and general social and assertive skills [46].

Programs formulated to combat the growing risk of substance abuse

The Ministry of Health and Family Welfare developed *Rashtriya Kishor Swasthya Karyakram* for teenagers aged 10 to 19, with a focus on improving nutrition, sexual and reproductive health, mental health, preventing injuries and violence, and preventing substance abuse. By enabling them to make informed and responsible decisions about their health and well-being and ensuring that they have access to the tools and assistance they need, the program seeks to enable all adolescents in India in realizing their full potential [47].

For the past six years, ‘Nasha Mukti Kendra’ in India and rehabilitation have worked to improve lives and provide treatment for those who abuse alcohol and other drugs. They provide cost-effective and dedicated therapy programs for all parts of society. Patients come to them from all around the nation. Despite having appropriate programs and therapies that can effectively treat the disorder, they do not employ medication to treat addiction.

## Conclusions

Around the world, adolescent drug and alcohol addiction has significantly increased morbidity and mortality. The menace of drugs and alcohol has been woven deep into the fabric of society. As its effects reach our youth, India's current generation is at high stake for the risk associated with the abuse of drugs like cannabis, alcohol, and tobacco. Even though the issue of substance abuse is complicated and pervasive, various stakeholders like healthcare professionals, community leaders, and educational institutions have access to a wealth of evidence-based research that can assist them to adopt interventions that can lower rates of teenage substance misuse. It is realized that while this problem is not specific to any one country or culture, individual remedies might not always be beneficial. Due to the unacceptably high rate of drug abuse that is wreaking havoc on humanity, a strategy for addressing modifiable risk factors is crucial. Because human psychology and mental health influence the choices the youth make related to their indulgence in drug misuse, it is the need of the hour to give serious consideration to measures like generating awareness, counseling, student guidance cells, positive parenting, etc., across the world. It will take time to change this substance misuse behavior, but the more effort we put into it, the greater the reward we will reap.
